# Nanopore sequencing elucidates *in vivo* development of meropenem resistance by insertion of a mobile genetic element in the porin gene *ompC* in *E. coli*

**DOI:** 10.1099/acmi.0.000757.v3

**Published:** 2024-02-06

**Authors:** Sébastien Matamoros, Jarne van Hattem

**Affiliations:** ^1^​ Department of Medical Microbiology and Infection Prevention, Amsterdam University Medical Centers, University of Amsterdam, Amsterdam, Netherlands

**Keywords:** antimicrobial resistance, whole genome sequencing, mobile genetic elements, insertion sequence

## Abstract

An ESBL-producing *E. coli* isolate recovered from a patient undergoing long-term treatment developed resistance to meropenem without acquiring carbapenem-hydrolysing enzymes. We performed Nanopore and Illumina sequencing and subsequent full hybrid genome assembly of this isolate and the meropenem-susceptible isolate recovered almost 8 weeks prior. Whole genome MLST patterns did not differ between isolates. However, we found the insertion of an IS5-like element in the sequence of the *ompC* gene and an increase in the number of copies of the CTX-M-15 gene in the resistant isolate. These results show that *E. coli* can develop meropenem resistance under antibiotic pressure by mutations in *ompC* genes and increasing the copy number of ESBL genes, and the value of next generation sequencing to reveal resistance mechanisms not detected by conventional PCR.

## Case presentation

A patient with a history of unresectable perihilar cholangiocarcinoma was transferred to our tertiary hospital with cholangitis and liver abscesses from a secondary hospital. An ESBL-producing *Escherichia coli* isolate recovered from blood cultures almost 8 weeks prior to the transfer (isolate A) showed no meropenem resistance (MIC 0.047 mg l^−1^) (Table 1). Under long-term treatment with meropenem and vancomycin, this patient developed fever and shaking chills. Blood cultures performed at our hospital showed growth of an extended-spectrum β-lactamase (ESBL)-producing *E. coli* (isolate B) that was meropenem resistant with a minimum inhibitory concentration (MIC) of 16 mg l^−1^ as determined by gradient test (Liofilchem), whereas the strain retained imipenem-susceptibility (MIC 1.5 mg l^−1^). No carbapenemase activity was detected by carbapenemase inactivation method [1] and no IMP, KPC, NDM, OXA-48 or VIM carbapenemase genes by qPCR. We stopped meropenem and started treatment with cotrimoxazole.

**Table 1.** Susceptibility profiles of the *E. coli* isolates

	Isolate A		Isolate B	
Date of isolation	24-4-2022		11-6-2022	
Antibiotic	Classification	MIC (mg l^−1^)	Classification	MIC (mg l^−1^)
Amoxicillin	R	>16	R	>16
Amoxicillin/clavulanic acid	R	>16	R	>16
Piperacillin/tazobactam	S	8	R	>64
Cefuroxime	R	>32	R	>32
Cefotaxime	R	>32	R	>32
Ceftazidime	R	32	R	>32
Ceftazidime/avibactam		nd	S	1*
Cefoxitin	S	≤4	R	32
Ertapenem		nd	R	>32*
Imipenem	S	≤0.25	S	1.5*
Meropenem	S	0.047*	R	16*
Cotrimoxazole	S	≤20	S	≤20
Trimethoprim	S	≤0.5	S	≤0.5
Ciprofloxacin	R	>2	R	>2
Colistin	S	≤0.5	S	≤0.5
Gentamicin	R	>8	R	>8
Tobramycin	R	>8	R	>8
Fosfomycin	S	≤16	S	≤16
Nitrofurantoin	S	≤16	S	≤16

Antibiotic MICs were measured with the automated susceptibility testing system Vitek 2 (bioMérieux) except those with *, by gradient strip (Liofilchem). EUCAST: Clinical breakpoints and dosing of antibiotics v 13.1 was used Interpretation of MICs for classification of susceptibility. Grey background indicates where a difference in susceptibility between both isolates was observed.

MIC, minimum inhibitory concentration; nd, not detemined R, resistant; S, susceptible.

Both isolates were subjected to Illumina whole genome sequencing, multi locus sequence typing (MLST) and whole genome MLST (wgMLST) using Bionumerics 8 (Applied Maths, Belgium) and AMR genes detection using ResFinder 4.0 [2]. Both isolates belonged to ST131 and had the same wgMLST profile (<10 alleles difference). Bioinformatics analyses of the whole genomes did not reveal in isolate B any additional resistance genes or other (point) mutations known to confer carbapenem resistance such as polymorphisms in genes *gyrA*, *parC* or others.

Mutations, deletions and insertions in the gene sequences of OmpC and OmpF porine proteins can increase carbapenem MIC’s by decreasing the drug influx in the bacterial cell [3]. To analyse this mechanism in our isolates, we performed genome assembly using SKESA [4] after removing low quality reads with Trimmomatic v0.38 [5]. A full sequence of the *ompC* gene could be retrieved from the genome assembly of isolate A, but the gene sequence of isolate B was located on two different contigs. Due to the nature of short reads genome assembly, this difference could be due to a bioinformatics artefact or a biological reason such as a recombination or insertion. We found no other difference in the sequence of the *ompC* gene.

Additionally, we sequenced both isolates with Oxford Nanopore technology using R9.4 Flow Cell on a MinIon following manufacturer’s instructions. We performed trimming of Nanopore reads for quality using NanoFilt [6] and hybrid assembly using both Illumina and Nanopore reads using UniCycler [7].

**Fig. 1.**
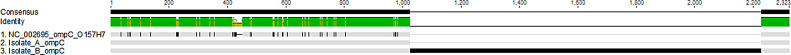
Alignment of the *ompC* genes from isolates A and B shows a 1200 bp insertion. Sequence NC 002695 is used as a reference for the position of the alignment. Grey sections of the alignment indicate 100 % similarity between the sequences; black sections indicate discrepancies.

Complete assembly of a circular chromosome and several plasmids was achieved for both isolates. Isolate A has a 5 111 702 bp chromosome and four plasmids (112 kbp; 53 kbp; 4 kbp and 3 kbp). Isolate B has a 5 129 626 bp chromosome and four similar plasmids with some insertions (112 kbp; 51 kbp; 8 kbp; 3 kbp). ResFinder revealed that isolate B possessed three copies of the CTX-M-15 gene compared to one for isolate A. One additional copy was located on the chromosome and the second one on the 8 kbp plasmid. When aligning the *ompC* genes from isolates A and B with that from another *E. coli* as reference (accession number NC_002695), the alignments revealed a 1200 bp insertion starting at nucleotide position 1026 (relative to the start of sequence in the sequence NC_002695) of the *ompC* gene of isolate B (Fig. 1).

We extracted the insertion sequence (IS), translated it into amino acids and compared to the NCBI non-redundant protein sequence database using Blast-P. It revealed a high identity between our sequence and an IS5-like element of the IS*Ec68* transposase family. IS elements including IS*Ec68* have previously been described as disrupting the sequence of *ompK36* gene, the *K. pneumoniae* homolog of *ompC,* and thereby elevating MICs of β-lactam antibiotics [8, 9]. *In vitro* studies showed porin-deficient subpopulations emerged in ESBL-producing *E. coli* during exposure to ertapenem at concentrations simulating human pharmacokinetics [10]. Additionally, IS26-mediated mechanisms underlying *bla*_OXA-1_ and *bla*_CTX-M-1_ group β-lactamase gene amplification with concurrent outer membrane porin disruption was found to lead to carbapenem resistance [11]. To our knowledge however, this is the first publication describing the *in vivo* evolution of this mechanism in *E. coli* isolates. Although we have not verified the level of expression, it has been shown previously that disruptions in the sequence of the *ompC* gene such as by insertion of IS elements leads to a significantly reduced expression of the OmpC protein [12]. These sequence disruptions also lead to a loss of fitness compared to the wild-type strains, but both the loss of fitness and the antimicrobial resistance traits can be reversed by complementing the mutants strains with *ompC*-carrying plasmids [13]. Additional experiments are needed to determine if the clinical isolates from the present study behave in a similar way.

Antimicrobial resistance in potentially pathogenic bacteria such as *E. coli* ST131 is a serious threat to hospitalized patients. In the present case, the carbapenemase resistance mechanism is probably porine deficiency combined with amplification of CTX-M-15 ESBL gene and arose in less than 8 weeks while under antibiotic treatment. No resistance determinant was detected by our qPCR method. We show that Nanopore sequencing is a valuable addition to the clinical microbiology laboratory to investigate the mechanism of unusual antibiotic resistance patterns in *E. coli*.
